# Direct imaging of uncoated biological samples enables correlation of super-resolution and electron microscopy data

**DOI:** 10.1038/s41598-018-29970-x

**Published:** 2018-08-02

**Authors:** José María Mateos, Gery Barmettler, Jana Doehner, Andres Kaech, Urs Ziegler

**Affiliations:** Center for Microscopy and Image Analysis, University of Zurich, Zurich, Switzerland

## Abstract

A simple method for imaging biological tissue samples by electron microscopy and its correlation with super-resolution light microscopy is presented. This room temperature protocol, based on protecting thin biological specimens with methylcellulose and imaging with low voltage scanning electron microscopy, circumvents complex classical electron microscopy sample preparation steps requiring dehydration, resin embedding and use of contrast agents. This technique facilitates visualization of subcellular structures e.g. synaptic clefts and synaptic vesicles in mouse brain tissue and the organization of mitochondrial cristae in the zebrafish retina. Application of immunogold protocols to these samples can determine the precise localization of synaptic proteins and, in combination with super-resolution light microscopy methods clearly pinpoints the subcellular distribution of several proteins in the tissue. The simplicity of the method, including section collection on a silicon wafer, reduces artefacts and correlates protein location with sample morphology.

## Introduction

Native-state preservation of the sample is the most critical factor to take into account when imaging at nanometer resolution^1,2^. Electron microscopy provides this nanometer range resolution but biological samples need to be prepared with elaborate protocols to resist the requisite high vacuum^3^. Dehydration is the most common method used to prepare samples for these demanding physical conditions. Water removal can however cause shrinkage and introduce extraction artefacts^4,5^. Cryo-preparation and imaging protocols provide excellent sample preservation; they are however, a challenging, laborious and expensive approach for samples larger than cells or small organisms^6^. Recently, the application of a graphene layer to the sample has been shown to protect cells from dehydration and vacuum^7^. Alternatively, Tokuyasu cryo-section protocol, a well-established method, uses methylcellulose (MC) to protect the sample from the high vacuum in the electron microscope avoiding the steps of dehydration and resin embedding or critical point drying^8,9^.

Contrast enhancement is another important requirement for imaging biological samples. Fluorescence microscopy provides an excellent means to differentiate signal from background but requires some counterstaining to obtain contextual information. Electron microscopy uses heavy metals such as uranyl acetate or osmium tetroxide to generate contrast from biological samples. Unfortunately, these steps compromise the antigenicity and fluorescence of the sample. Experiments requiring protein localization at subcellular resolution must develop protocols to circumvent these limitations. Several correlative methods allow combining fluorescence signals with *in situ* structural information^10–12^. We recently demonstrated that platinum shadowing can generate topographic contrast on thin Tokuyasu sections and combined it with immunogold or super-resolution microscopy^13,14^. However, in many cases, fine structures in complex samples like the synaptic elements (synaptic cleft, vesicles) in the central nervous system are difficult to detect.

We have developed a simple method which takes the advantages of the reported methods^13,14^, collection of Tokuyasu ultrathin sections on wafers and correlation of light and electron microscopy and now eliminated any additional source of external contrast agent. Sample’s contrast relies simply on the pure ultrastructural topography of the methylcellulose-embedded thin section imaged by low voltage scanning electron microscopy (LVSEM). LVSEM increases the topographic contrast of the sample^15^ and has been successfully used for strongly stained samples^16^ as well as for completely untreated samples like bacteria^17^ or cells^18^.

This method provides fine ultrastructural details, reduces the time required for preparation and minimizes possible artefacts by decreasing the number of preparation steps.

## Results

### Methylcellulose as unique medium for imaging with scanning electron microscopy (SEM)

**Figure 1:**
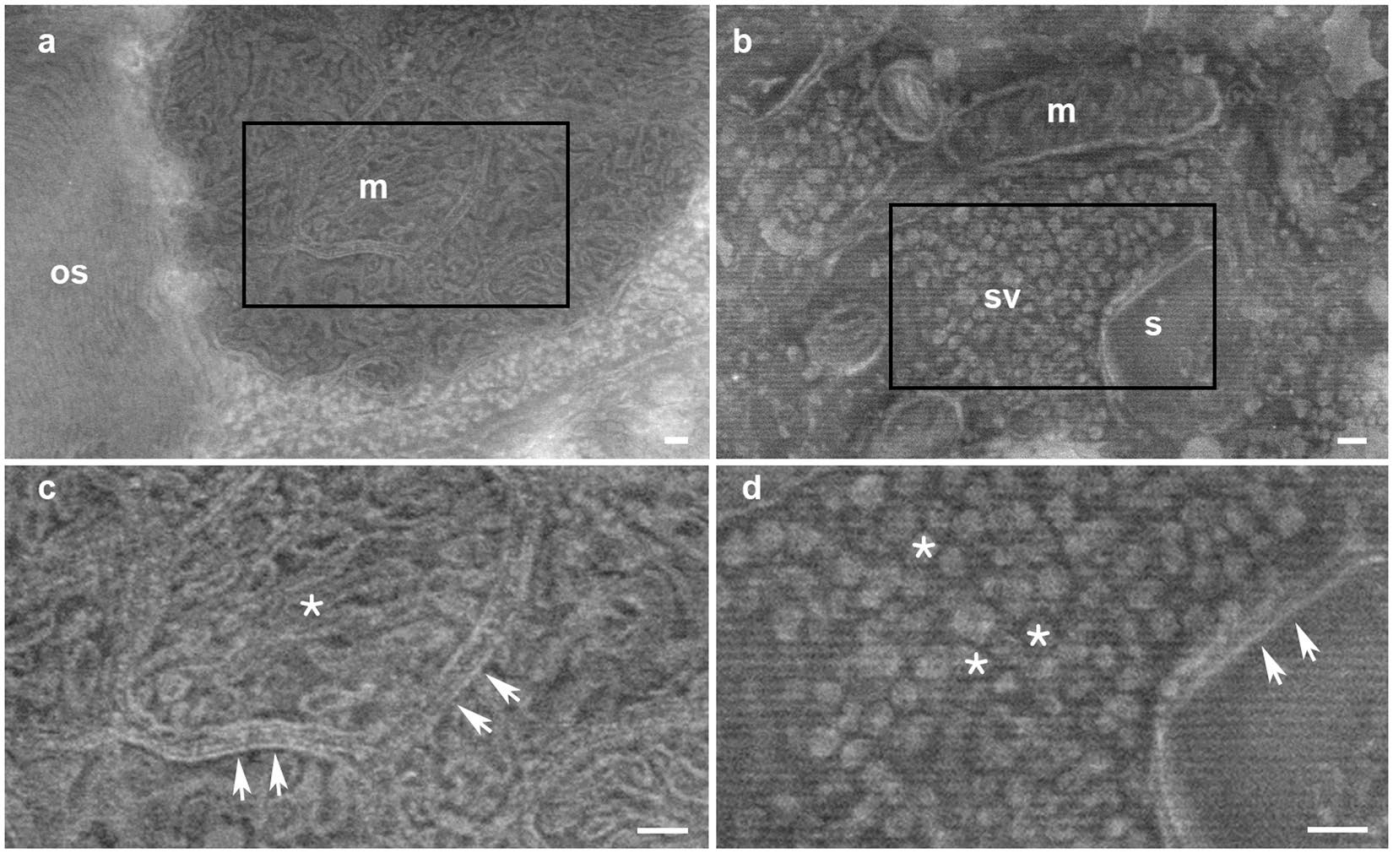
Uncoated-unstained biological samples imaged by low voltage scanning electron microscopy. (**a**) Zebrafish retina outer segment (os) and mitochondrial cluster (m). (**b**) Neuropil area of mouse cerebellum. A dendritic spine (s) forming a synapse with a synaptic bouton characterized by the accumulation of synaptic vesicles (sv). Mitochondria are also clearly visible (m). (**c**) High magnification image of insert in a showing the complex mitochondrial cristae (asterisk) and the separation between different mitochondria (arrows). (**d**) High magnification image of insert in b showing accumulation of synaptic vesicles (asterisks) at the presynaptic bouton and a synaptic cleft (arrows). Scale bars: 100 nm.

We collected Tokuyasu ultrathin sections on silicon wafers. After washing in PBS and fixation with glutaraldehyde (0.1% in PBS) we applied methylcellulose to the wafers, centrifuged them, dried at 40 °C for 10 minutes and brought directly to the scanning electron microscope. Low voltage (1.5 keV) imaging of the samples enabled identification of synaptic vesicles, synaptic clefts, mitochondria, presynaptic and postsynaptic membranes (Fig. 1). The method can also be used to investigate other organelles or applied to other tissues, such as nuclear pores, vacuoles or endoplasmic reticulum (Suppl. Figure 1). No heavy metal contrast agent was required to image fine ultrastructural details from samples.

**Figure 2:**
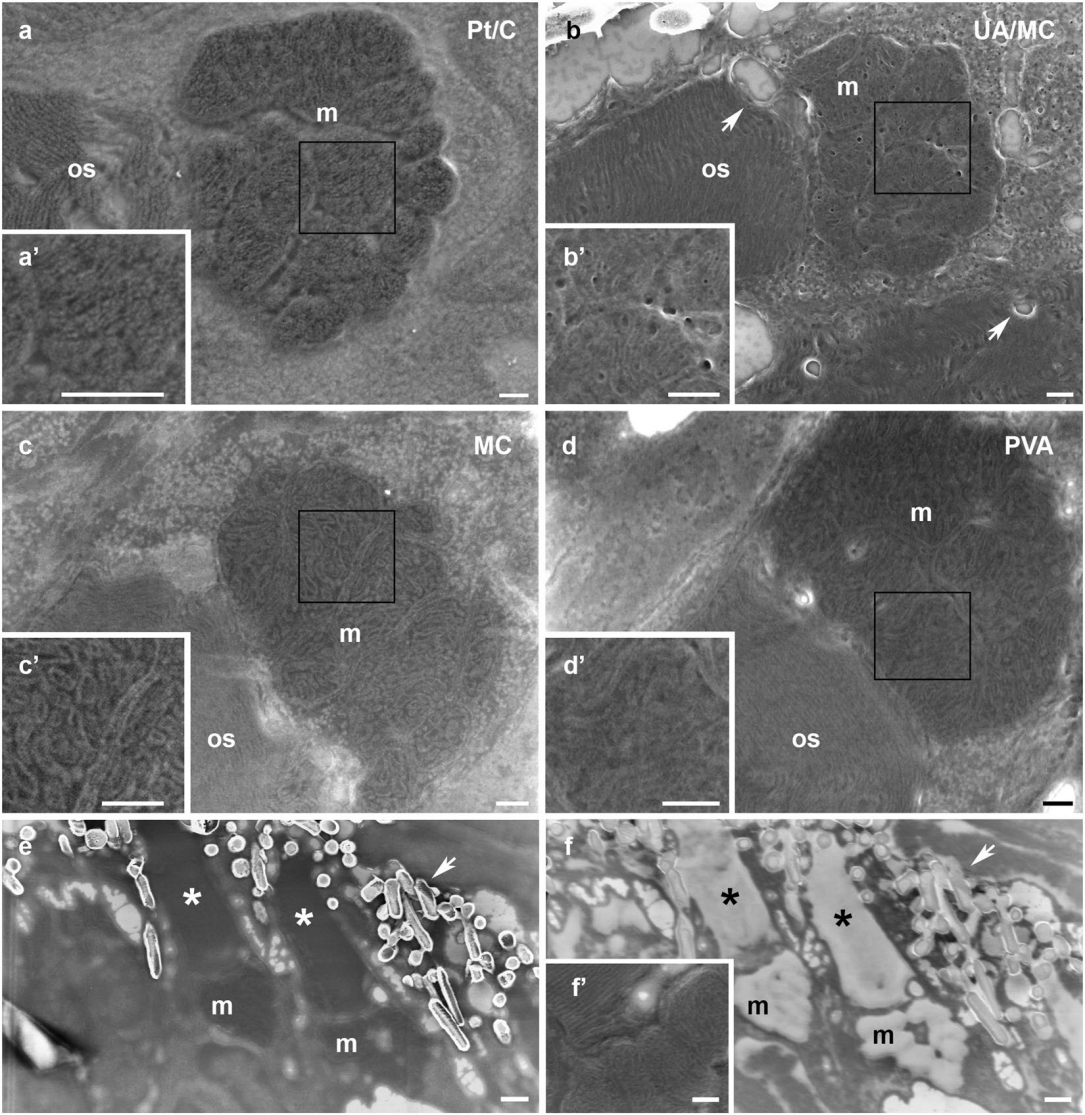
Comparison of contrast and resolution of biological membranes after different sample preparation protocols and with different acceleration voltage conditions. Mitochondria clusters (m) from zebrafish retina samples. (**a**) Sample coated with platinum/carbon (Pt/C; 10 nm) after MC treatment permits detection of membranes but, fine details as mitochondrial cristae are less well resolved as with the use of’ MC only. (a’) High magnification of insert in a. (**b**) Sections treated with MC and uranyl acetate (UA/MC; 3 mg/ml of 2% MC in water). Membranes are visible but, many small and large holes (arrows) appear in the tissue. (b’) High magnification of insert in b. (**c**) Section incubated with MC only (MC). Mitochondria cristae and membrane of the outer segment (os) are clearly visible. No signs of tissue damage are observed. (c’) High magnification of insert in c. (**d**) PVA is also a good tissue protectant for the SEM and similar results as with MC are obtained (PVA). (d’) High magnification of insert in d. (**e**) Zebrafish retina imaged at 1.5 keV. Outer segments (asterisks) and mitochondria (m) appear as dark elements. (**f**) Same sample imaged at 0.7 keV. Outer segments (asterisks and mitochondria (m) clusters appear as white elements at the beginning of the imaging. Melanin granules are labelled with an arrow. (f’) After scanning at low speed mitochondria membranes can be observed in high detail. Scale bars in a–d: 200 nm. Scale bars in inserts: 200 nm. Scale bars in e and f: 500 nm.

In order to evaluate the improvement in structural information and preservation of the sample treated with methylcellulose only, we compared these (Fig. 2) with other samples treated by the classical Tokuyasu method, contrasted with platinum shadowing^13,14^, or embedded with polyvinyl alcohol (PVA)^19^. Although platinum shadowing (Fig. 2a) enhanced stable imaging conditions in the scanning electron microscope, fine details such as small vesicles or membranes were not resolved. The classical Tokuyasu contrast method based on uranyl acetate staining during the methylcellulose step (Fig. 2b and Suppl. Figure 2) produced sufficient contrast but, to our surprise the tissue preservation was poor. We interpret that the pre-wash step in water applied before contrasting with uranyl acetate could lead to holes and disruptions of the tissue, possibly associated with the additional centrifugation of the wafer. Our initial experiments without any heavy metal contrast showed that the methylcellulose was reacting to the imaging. Image quality, contrast and resolved details improved during slow scans of the area of interest by probably reducing the thickness of the methylcellulose layer, most likely associated with increased curing by electron beam irradiation. We then dried the sample on the wafer before imaging at the SEM for 10 minutes at 40 °C on a heating plate to impart more stability during imaging. With these conditions, we demonstrate the imaging of synaptic elements (Fig. 1d) and mitochondria cristae (Fig. 2c). Similar results were obtained with PVA (Fig. 2d). In addition, we prepared classical Tokuyasu sections on EM grids and imaged them with a transmission electron microscope (Suppl. Figure 3). Ultrastructural fine details were observed in both methods.

### Effect of LVSEM imaging on the resolution and contrast of thin sections

Samples were individually scanned at different keV and analyzed the contrast and resolution for each condition to determine the imaging conditions for obtaining high contrast, high resolution and low charging for uncoated and non-conductive thin sections^20^ (Fig. 2). At 1.5 keV we observed dark areas like the mitochondria clusters or the outer membrane segment. The cytoplasmic areas of the sample appeared brighter and strong bright signals were obtained from the melanin granules (Fig. 2e). Interestingly, at 0.7 keV those dark dense areas (mitochondria clusters and outer segments) appeared very bright (Fig. 2f) at the beginning of the imaging. Scanning at low dwell times led to a conversion of this bright homogeneous layer to dark areas with fine structures visible such as mitochondria cristae or membranes of the outer segments (Fig. 2f’).

### Immunogold labeling on non-contrasted Tokuyasu sections

Immunogold labelling is a valuable method to determine the precise localization of proteins^21^. Conventional methods require either pre-embedding or post-embedding methods. Basic questions like the localization of a protein in relation to the synaptic cleft are answered by these methods providing fundamental information, key to understanding the function of such protein. The small size of the synapses and the complexity of the synaptic circuitries makes a basic question like: “is the protein pre- or post-synaptically located?” an experimentally demanding task^10^.

**Figure 3:**
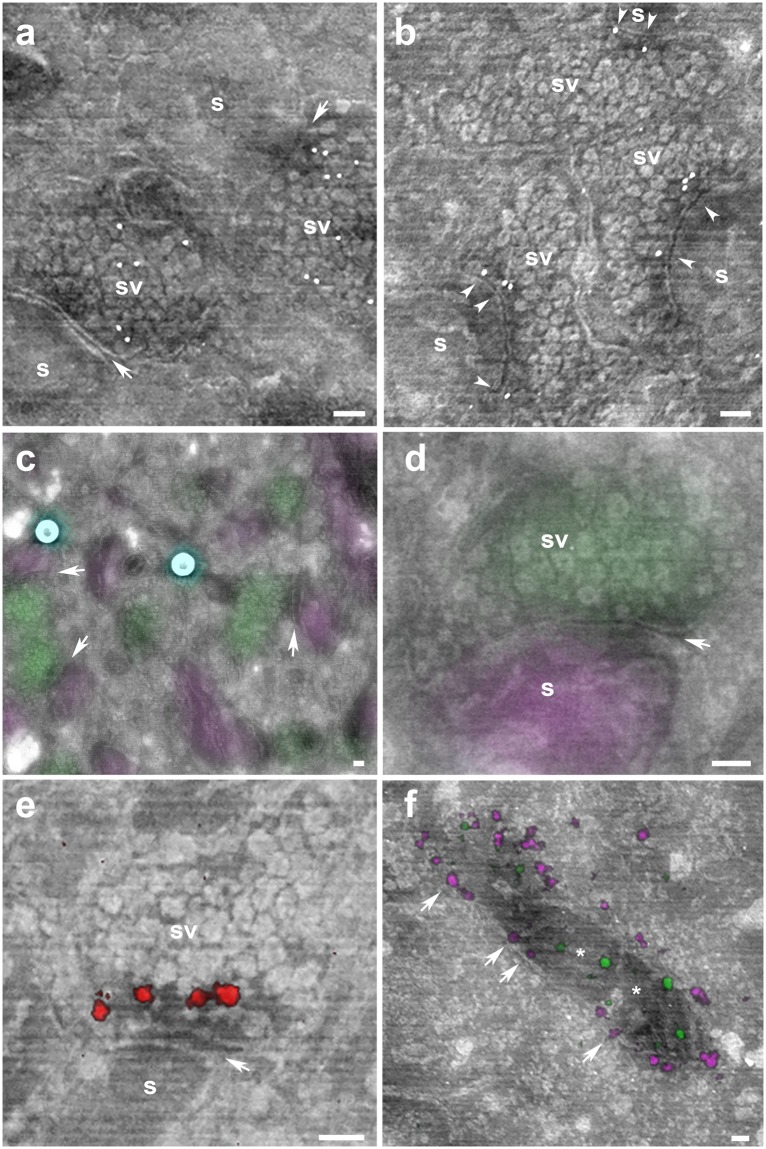
Single and double immunolabeling experiments on non-contrasted thin sections. (**a–b**) Immunogold synaptic protein localization on uncoated Tokuyasu sections. (**a**) Cerebellar synapses identified by the synaptic cleft (arrows), round dendritic spines (s) and the presence of many packed synaptic vesicles (sv) labeled with gold nanoparticles (white dots) for VGLUT1. (**b**) Bassoon immunopositive gold particles (white dots) are located at close proximity of the synaptic cleft (arrowheads) at the presynaptic side (sv). (**c**) Correlative confocal laser scanning microscopy and scanning electron microscopy shows the expression of the presynaptic protein VGLUT1 (green) and postsynaptic protein calbindin (magenta) in several cerebellar synapses (arrows). 170 nm fiducials (in image these appear as two round light cyan spheres) are used to align the light and electron microscopy images. (**d**) Many synaptic vesicles (sv) in a parallel fiber synaptic bouton stained with VGLUT1 (green) make a synaptic contact (arrow) with a dendritic spine (s) labeled with calbindin (magenta). (**e**) Super-resolution synaptic protein localization on non-contrasted Tokuyasu cerebellar sections. Synapse identified by the synaptic cleft (arrow) and the presence of many packed synaptic vesicles (sv) and a dendritic spine (s). Bassoon immunopositive signal (red) is located at close proximity of the synaptic cleft (arrow) at the presynaptic side (sv). (**f**) Dual colour GSDIM image of Tom20 (magenta) appearing at the outer membrane (arrows) of a mitochondria and the expression of ATP-Synthase (green) in the inner part of the mitochondria (asterisk) of Cos7 cells. All electron microscope images were acquired with a scanning electron microscope using an *in-lens* detector at an acceleration voltage of 1.5 keV. Scale bars: 100 nm.

We incubated Tokuyasu-sections on wafers with primary antibodies followed by secondary antibodies conjugated to gold nanoparticles and determined the localization of two well-known synaptic proteins, Bassoon, a protein of the presynaptic active zone^22^ and VGLUT1, a glutamate transporter in the membrane of synaptic vesicles^23,24^. Gold particles display the distribution of VGLUT1, appearing along the axonal terminal bouton (Fig. 3a). Bassoon distribution was much more precisely localized in the proximity of the synaptic cleft (Fig. 3b).

### Correlative light and electron microscopy

Conventional widefield or confocal laser scanning microscopy can be easily used for multicolor imaging^13,14^ (Fig. 3c,d). The advantage of using light microscopes is the large field of view either to determine protein expression or in finding rare events that are normally very demanding exercises in electron microscopy studies. After methylcellulose incubation and imaging in the scanning electron microscope, images can be easily correlated based on fluorescence signals (for example nuclei) and more accurately using fiducials (Fig. 3c). We used mouse cerebellum sections to localize synapses labelled with two synaptic proteins (VGLUT1 and calbindin) first by light microscopy when both signals appeared in close proximity; these structures were further elucidated by the presence of synaptic cleft on those putative synapses by electron microscopy (Fig. 3c,d).

### Correlative super-resolution and electron microscopy

Super-resolution microscopy achieves protein localization with nanometer-level resolution^25^. The use of ultra-thin sections (110 nm) has been demonstrated to be an ideal sample preparation for high precision in X, Y and Z dimension^25^. Correlation of super-resolution data with electron microscopy images allows the unequivocal identification of a protein in relation to the different organelles of the cells. We demonstrated the presynaptic expression of Bassoon at synaptic terminals of excitatory synapses by correlating the nanoscopic technique ground-state depletion and single-molecule return microscopy^26^ (GSDIM) with LVSEM images (Fig. 3e). No additional marker is necessary and the data is similar to those results obtained with immunogold, emphasizing that both methods can be well used for protein localization experiments. In addition, we performed two-colour GSDIM with thin sections of Cos7 cells to probe the ability of the method to detect two proteins, Tom20 and ATP-synthase each located at different compartments in the mitochondria^27^ (Fig. 3f).

## Discussion

Direct imaging of uncoated thin biological samples provides information about the topography of the sample. Fine structures like mitochondria cristae or synaptic vesicles are easily imaged and this contextual information can be combined with immunogold and/or super-resolution microscopy methods to determine, at nanoscale resolution, the location of a specific protein.

Low-voltage scanning electron microscopy resolves greater surface information, provides increased image contrast and minimizes charging effects of biological samples^20,28^, especially if samples are flat and thin^29^. In this respect, Monte Carlo simulations have been performed for back scattered electron (BSE) penetration and exiting depths in carbon at 1.5 keV to reach 80 nm and 14 nm, respectively^30^. Tokuyasu sections on a silicon wafer have a homogeneous thickness (ca. 110 nm) providing contrast from changes in density of the sample^31^ and this thickness, also improves the correlation in the Z- dimension of light and electron microscopy data.

Among the multiple correlative methods to determine protein localization at nanometer resolution^1,32–34^, those using Tokuyasu sections^11,13,14,25,35,36^ have several important advantages: antigenicity and fluorescent endogenous proteins are well preserved as well as the ultrastructure. It is possible to use cells as well as tissue. Those new methods in which the collection of the sections is made on hard support material such as coverslips or silicon wafers ease handling of the samples and additionally introduce stability in the imaging conditions. The present method can be applied to demonstrate, among others, the location of pre- or post-synaptic proteins in mouse tissue with high accuracy and good sample preservation. A main disadvantage of this method is the sectioning of those thin sections itself: specifically the difficulty of collecting consecutive sections, this can limit the method to mainly 2D approaches. In addition, careful handling of the frozen samples before cutting and during collection is crucial for good preservation of the ultrastructure of the sample.

Methylcellulose embedded cryo-sections collected on coverslips, contrasted with uranyl acetate have been demonstrated by Kopek and collaborators. They reported that the MC produced haziness during imaging and successfully exchanged MC with polyvinylalcohol (PVA)^11,35^. The limited level of details in fine structures like synaptic vesicles in brain tissue using the platinum shadowing method^13^ prompted us to improve the imaging conditions. Imaging uncoated samples at low keV and with long pixel dwell times (approx. 120 μs) revealed fine subcellular structures during the scan. We speculate that curing or thinning of the methylcellulose layer occurs by electron beam irradiation leading, at the same time, to the exposure of the biological surface and to a more detailed topographical signal. Drying the wafer for 10 minutes at 40 °C on a heating plate improved the imaging conditions and enhanced stability during imaging. Similar results were obtained with PVA suggesting that the limitation in the detection of fine details is the thickness of the embedding layer.

Counterstains, like DAPI or phalloidin, are often used in fluorescence microscopy to provide local morphological references of intra- and extracellular structures at the micrometre level. Super-resolution counterstains must however be chosen within the context of the nanoscale eg. a presynaptic counterstain for localizing a postsynaptic protein. The use of multiple counterstains for super-resolution combined with electron microscopy enables accurate localization of complementary affinity probes with nanoscale precision. In addition, the morphological information obtained by electron microscopy provides an assessment of the preservation of the sample in super-resolution experiments. We have developed a technique with thawed thin cryo-sections to access any part of an organ by sectioning, requiring simple equipment and most importantly achieving good tissue, as well as antigenicity preservation.

The simple application of methylcellulose to thawed cryo-sections and imaging under low-voltage scanning electron microscopy conditions provides a new way of imaging biological tissue without further steps, thus reducing possible artifacts associated with contrast and dehydration steps and allow quick correlation of multicolor super-resolution microscopy with scanning electron microscopy data.

## Methods

All animal experiments were conducted according to Swiss Laws and approved by the veterinary administration of the Canton of Zurich, Switzerland.

### Cryosectioning of mouse cerebellar tissue, zebrafish retina and cultured cells

Mouse tissue (2 animals) and cultured cells were prepared for Tokuyasu cryo-sectioning as described before^13^ and for zebrafish retina (3 animals) as described in Mateos *et al*.^14^.

Fluorescent beads (PS-Speck^TM^, #P7220, ThermoFischer, 1:3 in ethanol absolute) were used as fiducials to facilitate the correlation of the fluorescence and scanning electron microscopy images. Beads were added to the wafers before collecting the ultrathin sections. For an even bead distribution, they were air dried by placing an infra-red lamp (Philips InfraPhil 150 W) at a distance of 10 cm, immediately after adding the bead solution to the wafers.

### LVSEM imaging of uncoated and uncontrasted samples

Thin biological samples collected on silicon wafers were first washed on drops of PBS (2 × 10 min) at 0 °C in PBS. After washing the samples with PBS (3 × 1 min at RT) the wafers were post-fixed with 0.1% glutaraldehyde in PBS for 5 min followed by washes with PBS (2 × 2 min) and incubated with 2% methylcellulose in water at 0 °C (2 × 5 min). The wafers were then centrifuged at 14,100 × g for 90 s and immediately placed on a heating plate at 40 °C for 10 min. They were then mounted on an SEM aluminium stub with conducting carbon cement (Conductive carbon, Plano) and imaged in a scanning electron microscope (Zeiss Auriga 40) using an In Lens detector, pixel dwell time 120 us, at 5 mm working distance, and at an acceleration voltage of 1.5 keV (or 0.7 keV as in Fig. 2f).

To compare different contrast conditions, sections were either treated as above or: (1) contrasted with platinum/carbon by rotary shadowing^13,14^, (2) contrasted by incubation with MC and uranyl acetate (3 mg/ml of 2% MC in water) followed by centrifugation. This procedure includes an additional pre-wash with water before incubation with MC and uranyl acetate; (3) incubated with polyvinyl alcohol followed by centrifugation.

### Immunolabelling

Sections collected on wafers were washed with PBS (0.1 M, pH 7.4, 0 °C) as above, followed by incubation with 0.15% glycine in PBS (3 × 1 min) and washed with PBS (3 × 1 min). After a 5 minutes pre-incubation with PBG (PBS with 0.5% Bovine serum albumin and 0.2% gelatine type B) wafers were incubated with primary antibodies: (VGLUT1 (1:250; synaptic System #135302); Bassoon (2 ug/ml; Abcam, ab82958); Calbindin (1:125; Swant #300), Tom20 (4 ug/ml; Santa Cruz, sc-11415); ATP-synthase, (1:125; Abcam, ab 5432) followed by 6 × 1 min and 1 × 5 min washes in PBG. The following secondary antibodies were incubated and applied: (1) for immunogold experiments 12 nm gold goat anti-rabbit (1:10 in PBG; JIR #111-205-144) and 10 nm goat anti-mouse (1:10 in PBG; Sigma #G7777); (2) for confocal experiments, donkey anti-mouse F(ab′)_2_ AlexaFluor647 (1:200; JIR #715-606-150) and goat anti-rabbit F(ab′)_2_ AlexaFluor568 (1:200; LifeTech, A21069); (3) for super-resolution experiments, donkey anti-mouse F(ab′)_2_ Alexa Fluor 647 (1:200; JIR #715-606-150) and goat anti-rabbit F(ab′)_2_ Alexa Fluor 555 (1:200; ThermoFisher, A21430) single and double labelling Bassoon and double labelling, Tom20-ATP-Synthase. After immunolabelling for light microscopy, sections were incubated with DAPI (4′,6-diamidino-2-phenylindole dihydrochloride, Sigma, 1:250 dilution) for 10 sec and washed 2 × 2 min in PBS.

### Confocal laser scanning microscopy

To avoid drying of the sample, the sections were incubated in a solution of glycerine (80%) and PBS (1:1) for 10 sec and then transferred with the section facing down to a glass bottom (thickness 170 um +/− 5 um) petri dish (Ibidi). A pipette was used to remove most of the liquid underneath the wafer in order to bring it as close as possible to the bottom of the petri dish.

Confocal laser scanning microscopy was performed on a Leica SP5 inverted microscope (Leica Microsystems) with a 63x/1.4 NA oil immersion objective. Image stacks (60 × 60 × 170 nm) were acquired with a pinhole closed to 0.7 Airy units. Deconvolution was accomplished with Huygens Professional software (Scientific Volume Imaging B.V.).

### GSDIM

For single colour GSDIM, wafers were placed on droplets of a 1:1 solution of glycerol (80%) and imaging buffer containing an oxygen scavenging system (200 mM Phosphate buffer containing 10% glucose, 0.5 mg/ml glucoseoxidase, 40 ug/ml catalase, 15 mM beta-Mercapto-ethylamine hydrochloride (MEA HCI), pH 8.0). For double labelling the wafers were placed on droplets of a mixture of 20% Vectashield – 80% TRIS-Glycerol (5% v/v 1 M TRIS pH 8.0 in Glycerol)^37^. A glass bottom Petri dish as described in the confocal microscopy section was used, for super-resolution microscopy in addition, silicon strips were added to stabilize the imaging.

Super-resolution imaging was performed on a Leica SR-GSD 3D microscope (Leica Microsystems) using a 160x/1.43 NA oil immersion objective (HC PL APO, Leica Microsystems). The system was equipped with 488 nm/300 mW, 532 nm/500 mW and 647 nm/500 mW continuous wave lasers and an EMCCD camera (iXon Ultra, DU-897U, Andor). Images of 180 × 180 pixels were then taken with an integration time of 7 ms in epifluorescence mode. A total of 30,000 frames were collected for each reconstruction. Image reconstruction and visualization via Gaussian fitting was performed with the LAS X software (Leica Microsystems), by applying a detection threshold of 30 to 40 photons for each channel and a rendering pixel size of 4 nm.

### Registration and alignment of the correlated images based on fluorescence beads

Fluorescence beads were registered in the light and electron microscopy images and used for the alignment of the images with TrackEM2^38^ within the open-source platform Fiji^39^. Figures were prepared with the software Adobe Photoshop (CC 2015, Adobe). For presentation purposes, fluorescence signals were segmented, transparency modified and background subtracted.

## Electronic supplementary material

Supplementary Figures
