# Supplementary material for: Direct imaging of uncoated biological samples enables correlation of super-resolution and electron microscopy data
Source: Sci Rep. 2018 Aug 2;8:11610. doi: 10.1038/s41598-018-29970-x (PMC6072772; doi:10.1038/s41598-018-29970-x)
Supplement: Supplementary file 1 — Supplementary Figures [file 41598_2018_29970_MOESM1_ESM.docx]

Direct imaging of uncoated biological samples enables correlation of super-resolution and electron microscopy data

José María Mateos; Gery Barmettler; Jana Doehner; Andres Kaech; Urs Ziegler

**

**

**Supplementary Figure 1. Vesicles, nuclear pores and endoplasmic reticulum organelles from uncoated-unstained biological samples imaged by low voltage scanning electron microscopy.** (**a**) Mouse kidney proximal tubule. Cell nucleus (n). A nuclear pore is marked with an arrow. Endocytic vacuoles (asterisk). (**b**) Zebrafish retina photoreceptor cell nucleus (n). Nuclear pores are labelled with arrows and the endoplasmic reticulum cisternae with an asterisk. Mitochondria cluster (m). Scales: 200 nm.

**

**

**Supplementary Figure 2. Large field-of-view images to compare contrast and resolution of biological samples after sample preparation by methylcellulose versus classical Tokuyasu contrast and imaged with LVSEM** (**a**) Retina samples embedded in methylcellulose and imaged by LVSEM exhibited less holes compared with those (**b**) treated with methylcellulose and uranyl acetate. (**c**) High magnification image of insert in (a). Outer segments (os) and mitochondria clusters (m) as well as the surrounding cytoplasm are well- preserved. (**d**) High magnification image of insert in (b). Holes of different sizes are observed in the sample (arrow). Outer segments (os) and mitochondria clusters (m). Scales: 500 nm.


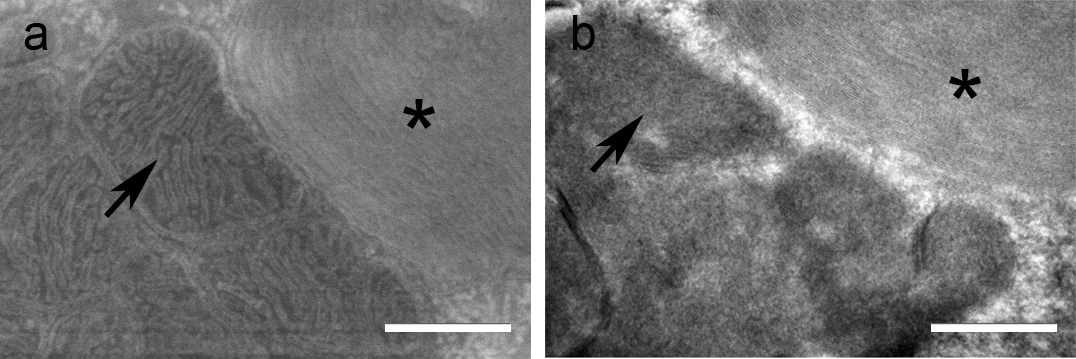


**Supplementary Figure 3. Comparison of uncoated zebrafish retina ultrathin sections processed by methylcellulose and LVSEM to classical Tokuyasu contrasted TEM sections.** (**a**) Uncoated zebrafish retina ultrathin section image acquired with a scanning electron microscope using an *in-lens* detector at an acceleration voltage of 1.5keV. Mitochondrial cisternae appear densely packed (arrow) and outer segment membranes closely aligned (asterisk). (**b**) Zebrafish retina ultrathin section processed by the classical Tokuyasu method (sections collected on an EM grid and contrasted with uranyl acetate/methylcellulose) and imaged with a transmission electron microscope (FEI Tecnai G2 Spirit). Scale bars: 500 nm.
